# Effect of network topology and node centrality on trading

**DOI:** 10.1038/s41598-020-68094-z

**Published:** 2020-07-06

**Authors:** Felipe Maciel Cardoso, Carlos Gracia-Lázaro, Frederic Moisan, Sanjeev Goyal, Ángel Sánchez, Yamir Moreno

**Affiliations:** 1 Institute for Biocomputation and Physics of Complex Systems, Universidad de Zaragoza, Zaragoza, Spain; 2 Unidad Mixta Interdisciplinar de Comportamiento y Complejidad Social (UMICCS), UC3M-UV-UZ, Madrid, Spain; 3 Department of Theoretical Physics, Faculty of Sciences, Universidad de Zaragoza, Zaragoza, Spain; 4 Faculty of Economics, Cambridge University, Cambridge, UK; 5 Faculty of Economics and Christ’s College, Cambridge University, Cambridge, UK; 6 Grupo Interdisciplinar de Sistemas Complejos, Departamento de Matemáticas, Universidad Carlos III de Madrid, 28911 Leganés, Madrid Spain; 7 Institute UC3M-BS for Financial Big Data (IBiDat), Universidad Carlos III de Madrid, 28903 Getafe, Madrid Spain; 8 ISI Foundation, Turin, Italy

**Keywords:** Statistical physics, thermodynamics and nonlinear dynamics, Complex networks

## Abstract

Global supply networks in agriculture, manufacturing, and services are a defining feature of the modern world. The efficiency and the distribution of surpluses across different parts of these networks depend on the choices of intermediaries. This paper conducts price formation experiments with human subjects located in large complex networks to develop a better understanding of the principles governing behavior. Our first experimental finding is that prices are larger and that trade is significantly less efficient in small-world networks as compared to random networks. Our second experimental finding is that location within a network is not an important determinant of pricing. An examination of the price dynamics suggests that traders on cheapest—and hence active—paths raise prices while those off these paths lower them. We construct an agent-based model (ABM) that embodies this rule of thumb. Simulations of this ABM yield macroscopic patterns consistent with the experimental findings. Finally, we extrapolate the ABM on to significantly larger random and small-world networks and find that network topology remains a key determinant of pricing and efficiency.

## Introduction

Globalization is a prominent feature of the modern economy^1^. Nowadays, supply, service and trading chains^2,3,3–6^ play a central role in different contexts such as agriculture^7–10^, transport and communication networks^11,12^, international trade^13^ and finance^14,15^. One key question on these systems is how pricing dynamics by intermediaries of the economy impacts both efficiency and surpluses. The purpose of this paper is to develop a better understanding of the forces that shape intermediary pricing behavior in such complex networks.

Game theory constitutes a useful framework to study competition among trading agents^16^. In this context, the Nash Bargaining Game^17^ studies how two agents share a surplus that they can jointly generate. In the Nash Bargaining Game, two players demand a portion of some good. If the total amount requested by both players is less than the total value of the good, both players get their request; otherwise, no player gets their request. There are many Nash equilibria in this game: any combination of demands whose sum is equal to the total value of the good constitutes a Nash equilibrium. There is also a Nash equilibrium where each player demands the entire value of the good^18^.

As a generalization of Nash demand game to *n* players, Choi et al.^19^ proposed and tested in the laboratory a model of intermediation pricing. In this model, a good is supposed to go from a source S to a destination D. Intermediaries, which are located in the nodes of a network, may post a price for the passage of the good. Trading occurs if there exists a path between S and D on which the sum of prices is smaller than or equal to the value of the good. The key finding was that the pricing and the surpluses of the intermediaries depends on the presence of “critical” nodes: a node is said to be critical if it lies on all possible paths between S and D. Condorelli and Galeotti^20^ provide an overview of the literature on intermediation and argue that the criticality of a node is an important determinant of pricing behavior, intermediation rents, and the efficiency of trading, in a wide class of models of auctions and bargaining. The goal of the present paper is to investigate this claim in large scale networks, so as to develop a better understanding of the role of network topology in commerce.

We conduct experiments with human subjects embedded in complex networks: specifically, we consider a random network and a small-world network each with 26 subjects (and the same level of average connectivity). In these networks there are *no* critical nodes: the results of Choi et al. would suggest that intermediary prices must be close to zero and that their surpluses must also be close to zero. As we will show below, our *first* finding is that, in all the networks studied, when there is not total but partial criticallity, intermediaries set positive prices and they make large profits. Moreover, network topology has powerful effects: in particular, in the random network, intermediaries set lower prices as compared to a small-world network. As a consequence, there is full trading efficiency in the random network, but trade breaks down in almost one third of the cases in the small-world network.

This striking difference leads us to an examination of how location within a network affects pricing: our *second* finding is that within a given network, standard measures of network centrality appear to have no significant effect on pricing behavior. As network location does not matter for prices, the presence on the cheapest and active path must be crucial for profits. And indeed, this is what we observe: intermediaries’ earnings are positively related to their betweenness weighted by the path length.

Turning to the dynamics of price setting, we observe that traders raise prices if they lie on the successful trade path (*i.e.*, the least-cost path), and that they lower prices when they are off the least-cost path. Based on these observations, we build an agent based (ABM) model that reproduces qualitatively the experimental results. We then use simulations to extrapolate our findings to larger networks: our *third* finding is that network topology continues to matter and that random networks exhibit lower prices and higher level of efficiency even when there are 100 traders. Finally, our *forth* finding uncovers the role of node-disjoint paths—two paths are disjoint if and only if they do not share any node-and of the average path length in shaping level of pricing in the simulations: networks that have a larger number of node-disjoint paths exhibit lower prices and higher efficiency. Among networks with the same number of node-disjoint paths, average path length is an important driver of costs.

## Experimental setup

We consider a simple game of price setting in networks to study supply, service and trading chains taken from Choi et al.^19^. Let ${\mathscr {N}}$ be a set of nodes ${\mathscr {N}}= \lbrace S, D, 1, 2, \ldots , n\rbrace$, where *S* is a source and *D* a destination; and ${\mathscr {L}}$ a set of pairs of elements of ${\mathscr {N}}$. ${\mathscr {N}}$ and ${\mathscr {L}}$ define a trading network where the elements of ${\mathscr {L}}$ are the links. A path between *S* and *D* is a sequence of distinct nodes $\lbrace i_1, i_2, ..., i_l \rbrace$ such that $\lbrace (S,i_1),(i_1,i_2), (i_2,i_3), \ldots , (i_{l-1},i_l),(i_l,D) \rbrace \subset {\mathscr {L}}$.

Each experiment consists of 4 series of 15 rounds each, and it involves *n* human subjects that will play the role of intermediaries. Before starting the first round of a series, each subject is randomly assigned to a node in $\lbrace 1, 2, \ldots , n\rbrace$. The positions of *S* and *D* are also assigned at random. These positions (players, *S* and *D*) remain constant over the 15 rounds. Subjects are always informed about the network and their position in it, that is, they can see the whole network including *S* and *D*. At each round, every subject has to make a decision; namely, she has to post a price from 0 to 100 tokens for the passage of a good by her node. The prices determine a total cost for every path between *S* and *D*. A path is feasible if its cost is not greater than a given threshold (100 tokens) that represents the value of the economic good generated by the path. After all players have made their choices, the cheapest path is selected: if it is feasible, each player located in this path receives her proposed price as a payoff. Otherwise, no trade takes place and payoffs are zero. Players who are out of the selected path do not get any payoff in that round. In the case of more than one cheapest path, the tie is resolved through a random choice among cheapest paths. From the second round onward, players are informed about the existence of a trade in the previous round, about the previously selected path, and about the prices and payoffs of all the players in the previous round together with their positions in the network.

We have conducted two experimental sessions in a random network of 26 nodes with $\langle k \rangle =3$ and two more sessions in a small-world-like network of 26 nodes with $\langle k \rangle =3$. Additionally, we have conducted another experimental session in a random network of 50 nodes with $\langle k \rangle =4$ that will allow us to check the robustness of the results against the size and connectivity of the network. These results can be found in SI sections 1–2 and SI Table S2. Further details can be found in Materials and Methods section.

## Results and discussion

**Figure 1:**
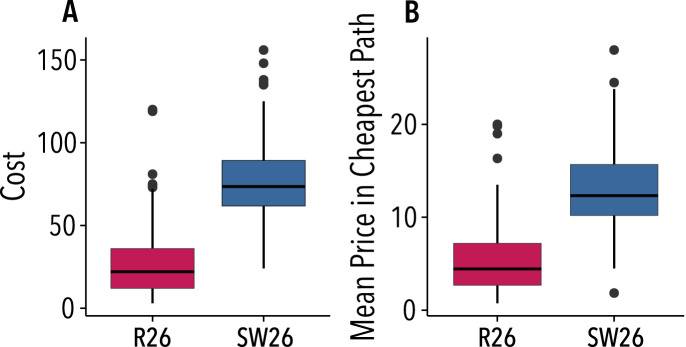
Network topology affects trading costs and prices. (**A**) Cost of the cheapest path for the random network of 26 nodes (R26), and for the small-world network of 26 nodes (SW26). (**B**) Mean price of participants in cheapest path for the same networks. Lines in the boxes denote the medians, whereas boxes extend to the lower- and upper-quartile values. Whiskers extend to the most extreme values within 1.5 interquartile range (IQR). Figure created using ggplot2 (v. 3.2.1)^21^.

The networks used in the experiment allow for coexisting paths with a different number of intermediaries, where theory predicts both efficient and inefficient (Nash) equilibria. Furthermore, these networks present different characteristics, such as degree and centrality distributions, that may affect the bargaining power of the intermediaries. These facts motivate our first question: How does the network topology affect costs and prices? Figure 1A shows the cheapest path cost in each of the networks considered. As shown, the small-world networks exhibit higher costs than random networks (t(232.41) = 15.5, $p<0.001$). Figure 1B instead displays the costs of the cheapest path normalized by the number of nodes on it, *i.e.*, the mean price of nodes along the cheapest path. The differences between networks persist, indicating that prices and costs strongly depend on the topology of the network. These results, separated by rounds, are shown in Fig. S4 of the SI. Table 1 shows that there is a very large effect of topology on efficiency: in the random network trade is realized in practically all the cases, while in the small-world network trade breaks down in almost one third of the cases (binomial-test, 0.95 CI=(0.76, 0.90), $p<0.001$). However, small-world networks involve higher costs and profits than random networks, since the higher posted prices compensate for the lower efficiency. Therefore, we conclude that the topology of the network matters for intermediation: the surpluses of the intermediaries vary significantly from one network class to the other in the experiments.

**Table 1:** Experimental results.

Network	Efficiency	Price	Price in CP	Cost	Profit	Length
R 26	0.97	11.34	5.49	28.33	1.10	6.26
SW 26	0.68	18.10	13.16	76.52	2.38	7.00

Efficiency (fraction of rounds in which the cheapest path cost was equal to or less than the threshold), and mean values of the price, price in the cheapest path, cost of the cheapest path, profit, and cheapest path length for the random network with 26 nodes (R 26) and for the small-world network with 26 nodes (SW 26).

**Figure 2:**
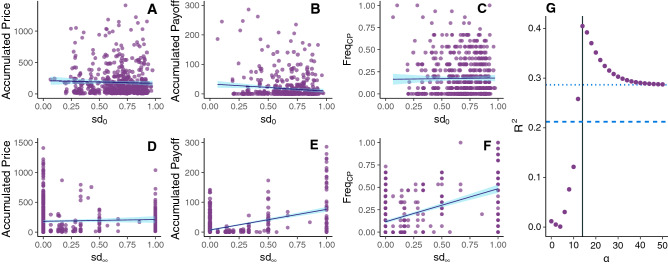
SD-betweenness determines payoffs but not posted prices. (**A–F**) Accumulated price (**A,D**), accumulated payoff (**B,E**) and frequency in the cheapest path (**C,F**) of participants during a series of 15 rounds as a function of the node criticallity $sd_0$ (**A–C**) and of the SD-betweenness $sd_\infty$ (**D–F**). (**G**) $R^2$ of the regression of participants accumulated payoff on $sd_\alpha$ versus $\alpha$, where $\alpha$ modulates the weight of the length of the paths in the S-D centrality measure. Dashed (points) line show the value of $R^2$ for correlation of payoffs on the betwenness (SD-betweenness). Data is pooled across any series of 15 rounds in any experimental session. For similar analyses within each experimental network, see SI Fig. S2. Figure created using ggplot2 (v.3.2.1)^21^ .

Profit is only obtained when the subjects are on the cheapest path, i.e., when they are on the path through which the trading is realized. Thus, it is of interest to examine what is the role of the location of intermediaries in the network in shaping their behavior, which we do next. First, we observe that the networks in our experiment do not contain any critical nodes and yet they generate large rents. So the results from the small scale experiments by Choi et al.^19^ do not apply to complex larger scale networks, the current study being an extension of work by Choi et al.^19^ to more complex networks. It seems likely then that nodes that are present on more paths have greater market power. This motivates a generalization of the notion of criticality as follows:

1 $$\begin{aligned} sd(v)=\frac{|P_{SD}(v)|}{|P_{SD}|} \;\;, \end{aligned}$$

where *sd*(*v*) is the *partial* criticality of node *v*, $|P_{SD}(v)|$ stands for the number of paths between the source and destination containing a given node *v*, and $|P_{SD}|$ for the total number of paths between the source and the destination. Following this line of thought, a higher partial criticality may indicate a potential for greater bargaining power and therefore nodes with a higher partial criticality should show higher prices and profits. Figure 2A shows the accumulated prices of the intermediaries as a function of their partial criticality. There is no significant relation between partial criticality and the prices posted by participants. Even more strikingly, as illustrated in Fig. 2B, there is no relationship between the accumulated payoff obtained and a node’s partial criticality. Figure 2C shows the frequency that each player is on the cheapest path versus her partial criticality. Again, there is no relation between these variables.

This lack of correlation may be due to the equal weighting of paths with different length. In order to address this point, we refine our generalized notion of partial criticality to take path length into account:

2 $$\begin{aligned} sd_\alpha (v)=\frac{\sum _{[S,v,D]} l(p)^{-\alpha }}{\sum _{[S,D]} l(p)^{-\alpha }} \; \; , \end{aligned}$$

where the summations are over all the paths between *S* and *D* containing *v* (numerator) and over all the paths between *S* and *D* (denominator). *l*(*p*) represents the length of path *p* and $\alpha$ stands for an arbitrary weight: as $\alpha$ increases, more importance is given to shorter paths. Specifically, when $\alpha \rightarrow \infty$ it will consider only the shortest paths, $sd_\infty (v)$ being a measure of the source-destination betweenness of node *v* (SD-betweenness(*v*)). On the opposite side, for $\alpha =0$ the partial criticality of Equation 1 is recovered, that is, $sd_0(v)=sd(v)$.

Figure 2D shows the accumulated prices of the intermediaries as a function of their SD-betweenness. Again, there is no relation observed between pricing behavior and betweenness. However, as shown in Fig. 2E, there is a positive correlation between the accumulated payoff obtained by intermediaries *v* and their $sd_\infty (v)$. That is, although pricing is uncorrelated with SD-betweenness centrality, profits are positively correlated with it. The reason behind this difference must therefore lie in how the presence of *v* on the least-cost path is correlated with $sd_\infty (v)$. This is displayed in Fig. 2F, which represents the fraction of times that an intermediary is on the cheapest path versus her SD-betweenness. As shown, there is a positive correlation between these measures, which explains why—in a situation where prices are largely insensitive to network location—profits will be correlated with $sd_\infty (v)$. The robustness of these results against the size and connectivity of the network is discussed in the SI, Section 2.A.

So far, we have seen that node centrality does not influence earnings when we equally consider all the paths from S to D to compute it, but it does when we consider only the shortest paths. This fact indicates that the weight given to paths length is important to study the capacity of the nodes to extract surpluses. In order to verify this hypothesis, Fig. 2G shows the coefficient of determination $R^2$ of the regression of intermediaries payoffs on $sd_\alpha$ as a function of $\alpha$. The best fit is obtained for $\alpha \sim 12$, which indicates that longer paths should have significantly smaller weight than shorter ones. As the number of paths grows exponentially with network size, SD-betweenness seems to be a feasible and good descriptor of participants’ earnings.

### Behavioral rules

We have noted that participants’ behavior is not determined by network position: criticality and classical measures of centrality are not good predictors of the prices posted by intermediaries. Nonetheless, results show differences in the prices posted by traders across different networks. Even if these networks might seem relatively small and similar, they are not. The environment (defined as the set of all the information that the individuals need to factor in their decisions) is very complex: there are many different paths passing through most of the traders, they need to take into account their price as well as those of other players, etc. It is thus reasonable to assume that the traders confronting such a complex and dynamic environment use rules of thumb, which on the other hand, should not depend on the network. In what follows, we develop a model that accounts for individual behavior and for the differences observed experimentally.

**Figure 3:**
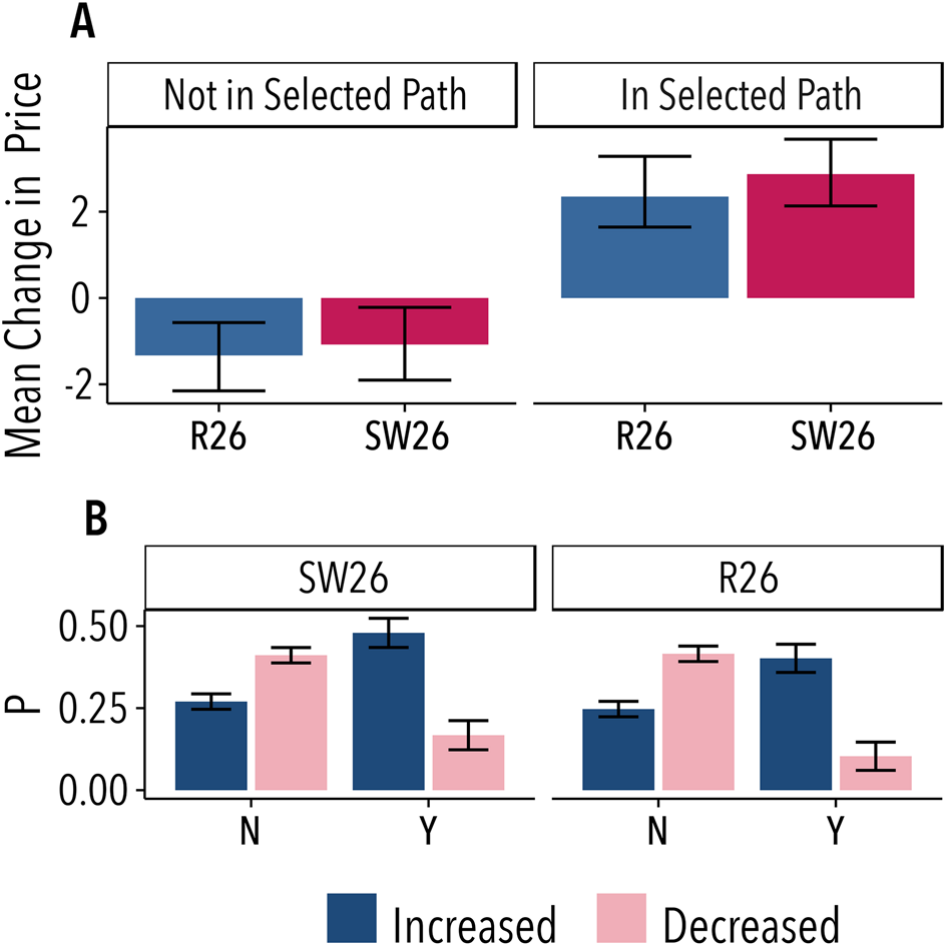
Being or not in the cheapest path determines the intermediaries price increases. (**A**) Mean changes in the posted price conditioned to have been (right) or not (left) in the selected cheapest path in the previous round for the random networks of 26 nodes (R26), and for the small-world network of 26 nodes (SW26). (**B**) Probability to increase (blue) and to decrease (pink) the posted price conditioned to have been (Y) or not (N) in the selected cheapest path, for each one of the studied networks. The error bars represent the 95% C.I. An extension of these results including the 50-nodes Random Network is displayed in SI Fig. S3. Figure created using ggplot2 (v.3.2.1)^21^.

Together with the network information, the other information shown to subjects is whether they were on the selected trading path. Figure 3A shows, for each one of the networks considered, the mean change in price for the cases when the participant was or was not along the cheapest path in the previous round. In the same way, Fig. 3B shows the probabilities to increase and to decrease the posted price conditioned to have been (Y) or not (N) in the cheapest path. Players appear to follow a simple rule, namely, to increase their price if they were on the cheapest path in the previous round and to decrease it otherwise. Furthermore, the expected values shown in Fig. 3A point out that successful intermediaries keep increasing their prices and therefore, without sufficient competition, costs and prices would always grow.

We now build a simple agent based model (ABM)^22^, as described below:

i) If node *u* belongs to a cheapest path at time *t*, it will change its posted price on time *t* + 1 by $\sigma$;
ii) If node *u* does not belong to a cheapest path at time t, it will change its posted price on time *t* + 1 by $-\rho$;
iii) The minimum price a node can post is 0.

**Table 2:** Numerical results in experimental networks.

Network	Efficiency	Price	Price in CP	Cost	Length
R 26	0.87	14.71	11.87	75.68	7.62
SW 26	0.66	15.14	12.56	94.32	8.97

Efficiency (fraction of rounds in which the cheapest path cost was equal to or less than the threshold), and mean values of the price, price in the cheapest path, cost of the cheapest path, and cheapest path length. Results obtained from numerical simulations with each one of the two studied networks with their corresponding source and destinations.

**Figure 4:**
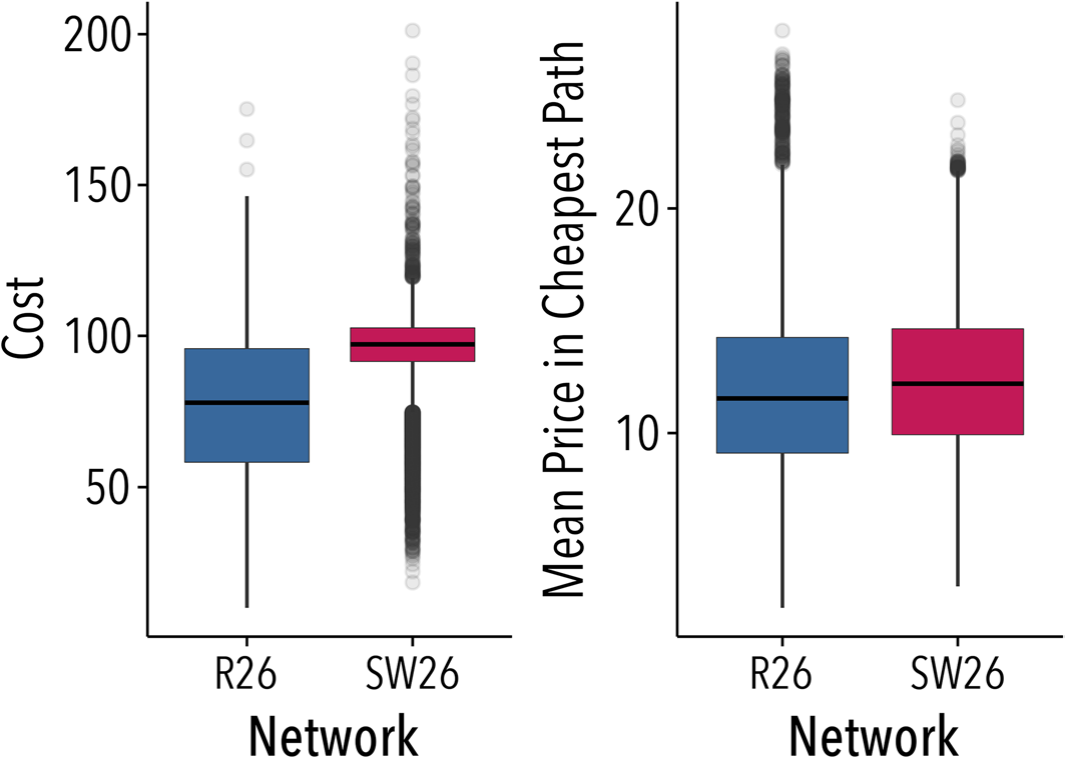
Numerical results of the model executed over the networks, source and destination from the experiments. Results shown are for 100 executions with 15 rounds for each network and source-destination pair, excluding the first round. Initial prices are bootstrapped from the experimental values. Values of $\sigma$ and $\rho$ are fixed and correspond to mean values of the experiment, respectively, 2.60 and 1.2. Figure created using ggplot2 (v.3.2.1)^21^.

To validate this model we executed it by bootstrapping the initial prices, the value of changes if on the cheapest path ($\sigma$) and the value of changes if not ($\rho$). The results, shown in Fig. 4, indicate that costs from simulations (resp. efficiency) are higher (resp. lower) in small-world networks than in random networks (t(9659.3)=68.33, $p<0.001$), in agreement with our experimental results. Costs reached relatively high values in some rounds, as the model does not incorporate participants direct response to the maximum cost threshold. Table 2 also confirms that topological differences between the networks are driving the differences in cost.

**Figure 5:**
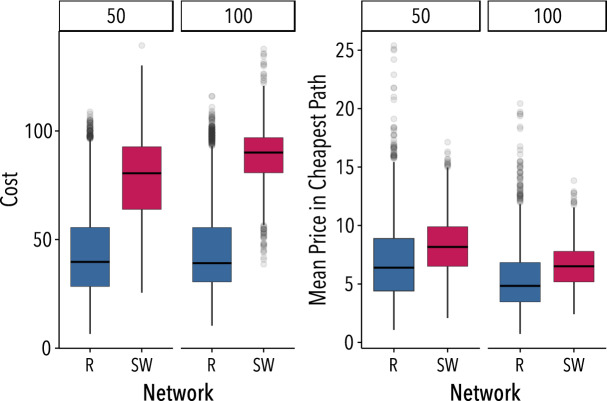
Numerical results of the model for networks with 50 and 100 nodes. Results shown are for 100 executions with 15 rounds for each network and source-destination pair, excluding the first round. Initial prices are bootstrapped from the experimental values. Values of $\sigma$ and $\rho$ are fixed and correspond to mean values of the experiment, respectively, 2.60 and 1.2. For similar analyses with random initial prices, see SI Fig. S7. Figure created using ggplot2 (v.3.2.1)^21^.

Once we have shown that the model captures very well the experimental observations, we verify if the same phenomena are observed in larger networks. Results for networks of size 50 and 100, shown in Fig. 5 and Table 3, are also consistent with the experimental data, confirming that the network topology has a significant effect on trading outcomes: small-worlds lead to higher costs and lower efficiency. A similar analysis with random initial prices, thus unlinking numerical results from those obtained from the experiments, can be found in SI section C3 and SI Fig. S7. Results in Fig. S7 are compatible with those shown in Fig. 5, providing more evidence about the effects of the network structure on prices and costs.

**Table 3:** Numerical results for larger networks.

Network	Efficiency	Price	Price in CP	Cost	Length
R 50	0.98	13.12	7.03	44.76	7.68
SW 50	0.91	14.32	8.28	77.44	10.74
R 100	0.97	12.65	5.53	46.50	10.05
SW 100	0.82	13.20	6.56	88.56	15.41

Efficiency (fraction of rounds in which the cheapest path cost was equal to or less than the threshold), and mean values of the price, price in the cheapest path, cost of the cheapest path, and cheapest path length. Results obtained from numerical simulations with random networks with 50 and 100 nodes (R 50, R 100) for the small-world network with 50 and 100 nodes (R50, R 100).

### Topological properties behind the differences in cost

Finally, we go one step further in order to explain what lies behind the differences found in costs. One possible theoretical hypothesis could be that costs depend on competition between paths. In our setup, this would be equivalent to assume that costs should decrease with the number of possible ways to reach the destination, *i.e.*, the number of independent (sets of) paths from S to D. Specifically, we expect competition to be proportional to the number *M* of node-disjoint paths^23^, as it captures the possible number of simultaneous independent trades (see SI Section II.C.1 for a deeper discussion on this subject). According to this hypothesis, the larger the value of *M*, the lower the cost. Another possible explanation for the dependency of costs with the networks could be the structural differences between the latter. It is well known that clustering coefficients and average path lengths differ for the SW and the random networks considered in our experiments ($p \in \{0.1, 1\}$^24^), and therefore the observed differences in cost could be tied to variations in those properties.

**Figure 6:**
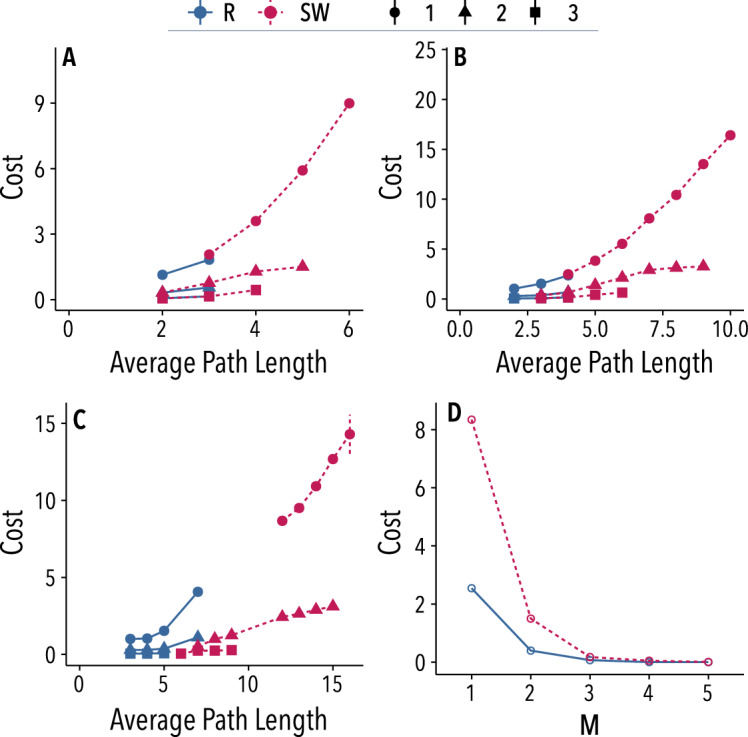
Numerical results of the model. (**A–C**) Average final cost (in $10^4$) of the cheapest path after a period of $10^4$ rounds as a function of the average path length of the network. Different panels correspond to different network sizes: 26 (**A**), 50 (**B**), and 1,000 (**C**) nodes; colors correspond to different network models: random (blue) and small-world (magenta); and different shapes correspond to different values of the number *M* of disjoint paths. For each configuration, there were generated 10,000 networks of size 26, 50, and 1,000, according to the Watts-Strogatz algorithm^24^ with *p* = 0.1, 1 and average degree from 2 to 10. The initial cost was set to 0 and the increment/decrement ratio was fixed to the experimental value ($\sigma /\rho =2.4$). Results for $M > 5$ are not shown as costs converge fast to 0. **D:** Mean value of the cost of the cheapest path versus *M* for the same networks. See the main text for further details. Figure created using ggplot2 (v.3.2.1)^21^.

In order to verify the previous hypotheses, we executed a version of the model without the maximum cost threshold. With this setup, we can study long-term effects after a sufficiently large number of rounds and uncover the cost tendency. In this regime, we cannot analyze network efficiency, however, networks yielding higher cost should be more inefficient. Note that the proposed model allows extrapolating the observed behavior to larger networks with a large range of values of *M*. Then, we can generalize the observed experimental results to larger networks, which allow us to find the (theoretically conjectured) influence of *M* on prices. We ran the algorithm for $10^4$ rounds and then we considered the final cost of the trade for each configuration. Results for networks of size 26, 50 and 1,000 nodes are shown in Fig. 6A–C, respectively. Simulations of trading dynamics on the aforementioned networks indicate that the number of node-disjoint paths (*M*) between *S* and *D* is the best indicator of final cost. Figure 6D shows that as *M* grows, the costs are reduced so drastically that they go to 0 for $M>3$. Moreover, the numerical results also reveal that for networks with the same value of *M*, the cost grows with the average path length. Indeed, this dependency explains why costs on small-world networks tend to be larger: these networks have a larger average path-length. To show that this finding is not a consequence of differences in the length of the cheapest paths, Fig. S5 of the SI displays, for the same simulations, the costs of the cheapest path normalized by the number of nodes on it versus the average path length of the network. It can be seen that the mean price of nodes in the cheapest path also correlates with the average path length. Interestingly, even though in this regime the difference in the clustering is larger than the difference in average path length, the former is not a good indicator of costs ($R^2=0.57$ vs. $R^2=0.79$, see Section 2.C.2, table S3, and Figure S6 in the SI). In summary, these results provide two stylized facts that may guide future inquiries in this line, namely, trading costs will be null in setups with a relatively large number of node-disjoint paths and costs should be larger in networks with larger average path length. Note that, although the influence of the type of network on efficiency, costs, and prices is an experimental result, the effect of both the number of disjoint paths and average path length on costs is a result obtained from the proposed model.

## Conclusions

Our experimental results indicate that the trading network has a powerful effect on both the pricing behavior of intermediaries and the overall efficiency of the system, random networks being more efficient and showing significantly lower prices than small-world networks. However, within a network, prices are relatively insensitive to node location, but intermediaries with greater betweenness make larger profits. Informed by the experimental results, we introduced an ABM of pricing behavior to understand traders’ pricing. The key input of the model is the experimental observation that intermediaries raise prices when they lie on the cheapest path and lower their prices otherwise. The model successfully reproduced qualitatively the experimental results and allowed us to extrapolate and anticipate outcomes of pricing and efficiency to scenarios involving larger networks and longer timescales. Important enough, the model also enabled the discovery of what are the key determinants of cost, namely, the number of node-disjoint paths from source to destination and the network average path length. Ultimately, this explained the differences in our experimental results: in a small-world network, the average path length tends to be larger and this leads to higher costs and lower efficiency of trading in these networks as compared to random networks.

Overall, our work reveals that the topology of trading networks is key to determine their efficiency and cost. It would be interesting to further test our conclusions using real data on trading, in particular, the finding that the availability of node-disjoint paths takes trading costs down. On the other hand, our insights may be useful for the design of competition-improved networks for goods currently overpriced due to intermediation. Further research on the role of information provided to intermediaries and on other network topologies will be also relevant to address these issues.

## Methods

We carried out 5 experimental sessions, each of which is composed of 4 consecutive series of 15 rounds each. All the networks have been generated through the Watts-Strogatz algorithm^24^ with different probabilities *p* of rewiring (*p* = 0.1 for small-world-like networks and *p* = 1 for random networks). Mean degree was $\langle k\rangle =3$ for the 26 nodes networks and $\langle k\rangle =4$ for the network of 50 nodes. A representation of each network as viewed by the participants is shown in SI Fig. S1. The experiment was conducted with 144 volunteers recruited from the volunteer pool of the IBSEN project (http://www.ibsen.eu). The first experimental session was performed on 29 June 2017, jointly at Experimental Economics Labs of University of Zaragoza (UZ) and University Carlos III of Madrid (UC3M), with a random network of 50 nodes. A second experimental session was performed on 31 October 2017, at UC3M, with a SW network network of 26 nodes. Subsequent three sessions were performed at UZ on 14 November 2018 (RN, 26 nodes), 24 April 2018 (RN, 26 nodes), and 25 April 2018 (SW, 26 nodes). Table S1 of the SI shows the demographic data of the sessions.

All the participants played through a web interface after reading a tutorial (both included in SI Materials and Methods) on the screen. When everybody had gone through the tutorial, the experiment began, lasting for approximately 90 minutes. At the end of the experiments, all participants received their earnings and their show-up fee. Total earnings in the experiment ranged from 5 to 82 Euros, average earning was 18.4 Euros.

All participants signed an informed consent to participate. Besides, their anonymity was always preserved (in agreement with the Spanish Law for Personal Data Protection) by assigning them randomly a username which identified them in the system. No association was ever made between their real names and the results. This procedure was checked and approved by the Clinical Research Ethical Committee of IACS, Aragon Government: accord 12/2017. All methods were performed in accordaxnce with the relevant guidelines and regulations.

## Supplementary information

Supplementary information

## Data Availability

The datasets analysed during the current study are available from the corresponding author on reasonable request.
