# Supplementary material for: Effect of network topology and node centrality on trading
Source: Sci Rep. 2020 Jul 6;10:11113. doi: 10.1038/s41598-020-68094-z (PMC7338384; doi:10.1038/s41598-020-68094-z)
Supplement: Supplementary file 1 — Supplementary information [file 41598_2020_68094_MOESM1_ESM.pdf]

Supplemental Materials:
Effect of network topology and node centrality on trading

Felipe Maciel Cardoso, Carlos Gracia-Lázaro, Frederic Moisan, Sanjeev Goyal, Ángel Sánchez, and Yamir Moreno

I. EXPERIMENTAL SETUP

A. Volunteers recruitment and experimental platform

The experiment was carried out with volunteers chosen among the pool of volunteers of the EU project IBSEN (ibsen-h2020.eu). This pool consists of more than 24,000 volunteers (24,210 at the time of the experiment) from different countries to perform social experiments. We restricted the call to volunteers residing in Madrid or Zaragoza (10,408 volunteers), where the experiments were performed. Following the call for participation, we selected 144 volunteers, whose demographic data are shown in Table S1. In order to satisfy ethical procedures, all personal data about the participants were anonymized and treated as confidential.

Network	Session	Number of participants	Mean age (SD)	Range age	women	City	Date
R 50	1	48	32.42 (11.84)	21-64	64.58%	Madrid and Zaragoza	29 JUN 2017
R 26	1	24	39.54 (12.50)	19-61	70.83%	Zaragoza	14 NOV 2017
R 26	2	24	34.29 (15.32)	20-66	62.50%	Zaragoza	24 APR 2018
SW 26	1	24	22.67 (2.39)	19-31	20.83%	Madrid	31 OCT 2017
SW 26	2	24	34.29 (15.32)	20-66	62.50%	Zaragoza	25 APR 2018
Aggregated		144	33.55 (13.76)	19-72	57.64%		

TABLE S1. **Experimental sessions**. Demographic data of the participants. R50 stands for a random network of 50 nodes, R26 for a random network of 26 nodes and SW26 for a small-world-like network of 26 nodes. For the R26 and SW26 networks, two experimental sessions were carried out. The R50 session was held jointly in Madrid and Zaragoza, the rest of sessions were performed either in Madrid or in Zaragoza.

The experiment was run using an application based on the oTree platform [S1]. At the beginning of each session, all participants were located at a desktop computer spot. When everybody had read the instructions, the first series began. Each series lasted 15 rounds approximately 15 minutes. At the end of the fourth series, the experiment finished and all participants received their earnings plus a show-up fee of 5 euros. Total earnings ranged from 5 to 82 euros (mean was 18.4), including the show-up fee, and the sessions lasted between 70 and 85 minutes (mean was 77), including instructions reading. The experiment is described in the section 'Experimental setup' of the main text. Both the sessions and the networks are described in the section 'Materials and methods' of the main text.

B. Description of networks

ll the networks used in the experiment are generated through the Watts-Strogatz [S2] algorithm with different probabilities p of rewiring ($p = 0.1$ for the small-world-like network and $p = 1$ for the random networks). In any given treatment (R26, SW26, and R50), the same network was used across all series and sessions. However, the selection of source-destination pairs is generated randomly at the beginning of each series such that the shortest path between the two nodes is of distance at least diameter - 2 (*resp.* - 1) for the R26 and SW26 (resp. R50) networks (as a means to prevent uninteresting scenarios with very short distance between source and destination nodes). Mean degree is 3 for the 26 nodes networks and 4 for the network of 50 nodes. Fig. S1 depicts the structural representation of the network as viewed by the subjects in each corresponding treatment: Random Network of 26 nodes (left), Small-World-Like Network of 26 nodes (center) and Random Network of 50 nodes (right).

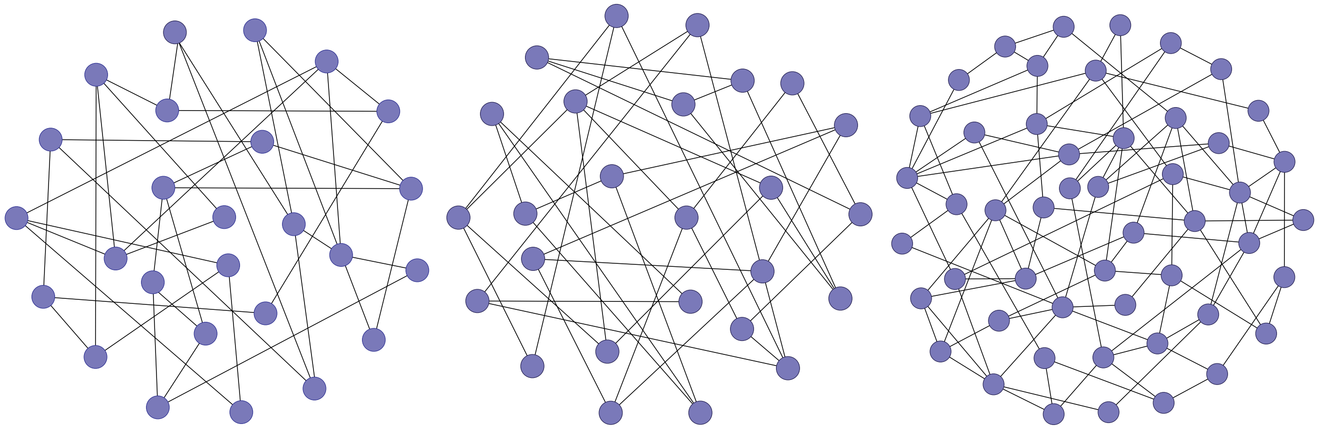

FIG. S1. Networks used in the experiments: 26-nodes Random Network (left), 26-nodes Small-World-Like network (center) and 50-nodes Random Network (right).

C. Instructions for players

Since there were three possible networks (random networks of 50 and 26 nodes and small-world-like network of 26 nodes), there were three different instructions that differentiated only in this single aspect. In what follows a translation of the original Spanish instructions (available upon request) for the 26-nodes random network is included.

Instructions

Thank you for participating in this experiment, that is part of a research project in which we try to understand how individuals make decisions. You re not expected to behave in any particular way. At this moment the experiment begins. Please keep quiet until the end, turn your cell phone off, and remember that the use of any material foreign to the experiment is not allowed (including pen, pencil or paper).

Your earnings will depend on your own decisions and those of the other participants. Additionally, you will receive 5 € for participating in the experiment until the end.

Please keep quiet during the experiment. If you need help, raise your hand and wait to be assisted. Please do not ask any question aloud.

You participate along with other people with whom you interact according to the rules explained below. The session lasts about an hour and a half. The following instructions are the same for every participant of this experiment.

Once completed the session, you will receive 5 € for participating, along with your earnings corresponding to the rounds, once converted into euros. For convenience, the total earnings are rounded up to the nearest 50 cents.

You will access the experiments after reading these instructions. When all participants have accessed, the rounds will begin.

You are going to participate in 4 experiments. Each experiment consists of 15 rounds. Before starting each experiment, all the players, you included, will be randomly located in the nodes of the network shown below. Your position in the network will be denoted with the letter '**M**' (for me). In the same way, two different nodes will be chosen as Source (**S**) and Destination (**D**) respectively. Their position in the network will be denoted with the letters '**S**' and '**D**'. **All the players will remain in the same position during each experiment of 15 rounds.** In the same way, the source and destination will remain in the same position throughout each experiment of 15 rounds. The players will play the role of intermediaries. A good must be transported from **S** to **D** generating a benefit of 100 tokens for all players involved (**S**, **D**, and all nodes in the path between them). Intermediaries (that is, the players) simultaneously have to post the fraction of these 100 tokens they would like to charge if selected, which must be between 0 and 100 tokens. **You will have 60 seconds to post your price.** If you do not post a price, the computer will decide for you: please do not run out your time and make your own decision.

This is the screen you will see in the first round (this screenshot is only an example):

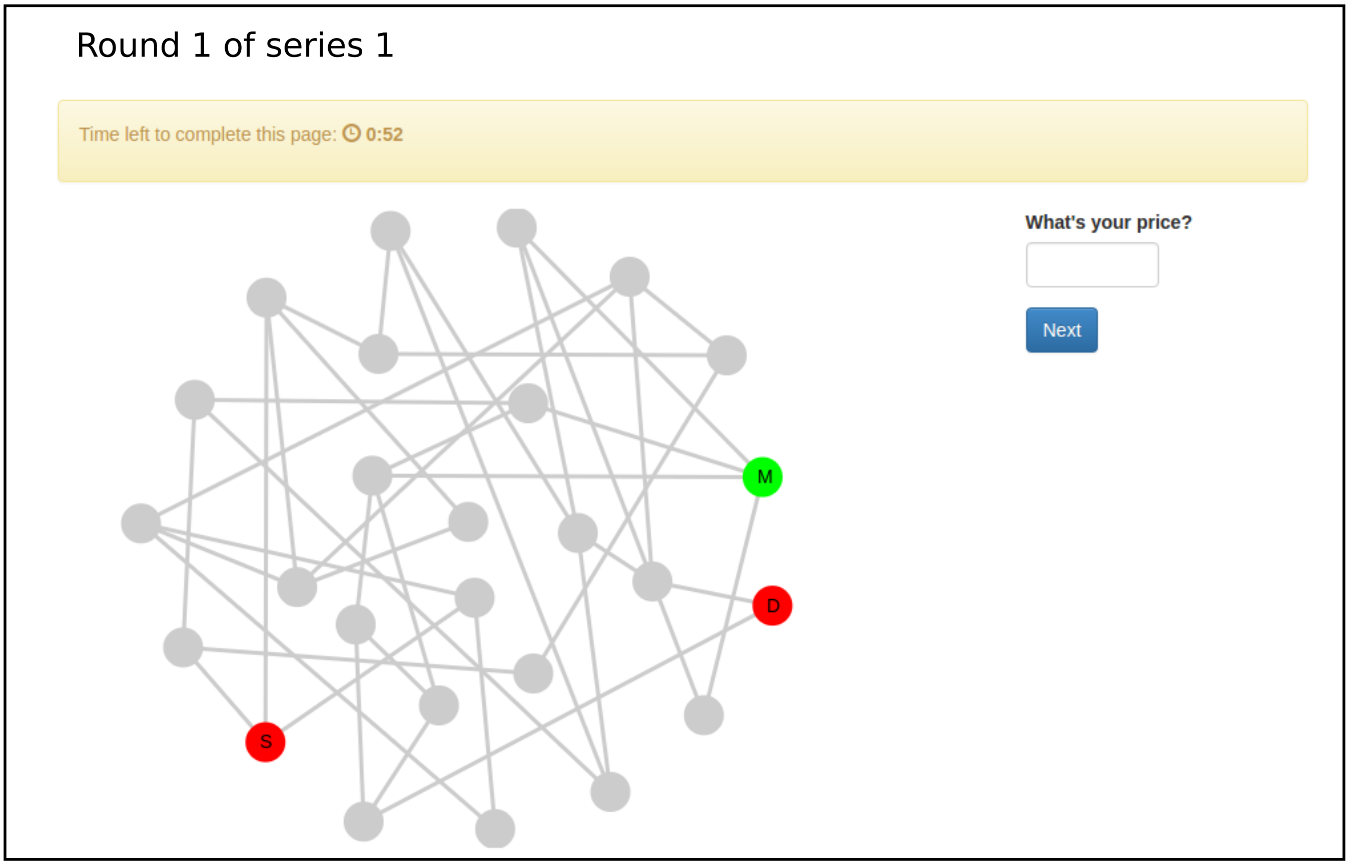

The sum of prices along any given path between **S** and **D** determine a total cost. Once all the intermediaries have posted a price, the cheapest path (with lowest total cost) from **S** to **D** will be selected. If the total cost of the cost cheapest path is less than or equal to 100 tokens, the good will be taken from **S** to **D**. Otherwise, that is, if all the paths from **S** to **D** cost more than 100 tokens, there will be no deal and no value will be generated. Ties are broken randomly, that is, if there are more than one cheapest path, one of them will be selected at random.

Your payoff in this round will be:

a) If you are located on the selected cheapest path, you will receive your price as payoff. b) Otherwise, that is, if you are not on the selected path, you will not receive any payoff in that round. S and D will receive, equally distributed, the rest of the 100 tokens.

From the second round on, you will be informed about whether there was a deal in the previous round, and if so what was the selected cheapest path, and the costs of this path. You will also be informed about the cheapest path through your node regardless of whether this was the selected cheapest path. The selected path will be highlighted by a dashed red line, while the cheapest path through your node will be highlighted by a blue solid line. Note that the cheapest path through your node may contain loops, i.e., it may pass more than one time through some nodes. With this information on the screen, you must set a price for the current round. At the end of each experiment, the positions of all players, the source, and the destination will be randomly reassigned, and a new experiment of 15 rounds will begin.

This is the screen you will see in the subsequent rounds (this screenshot is only an example):

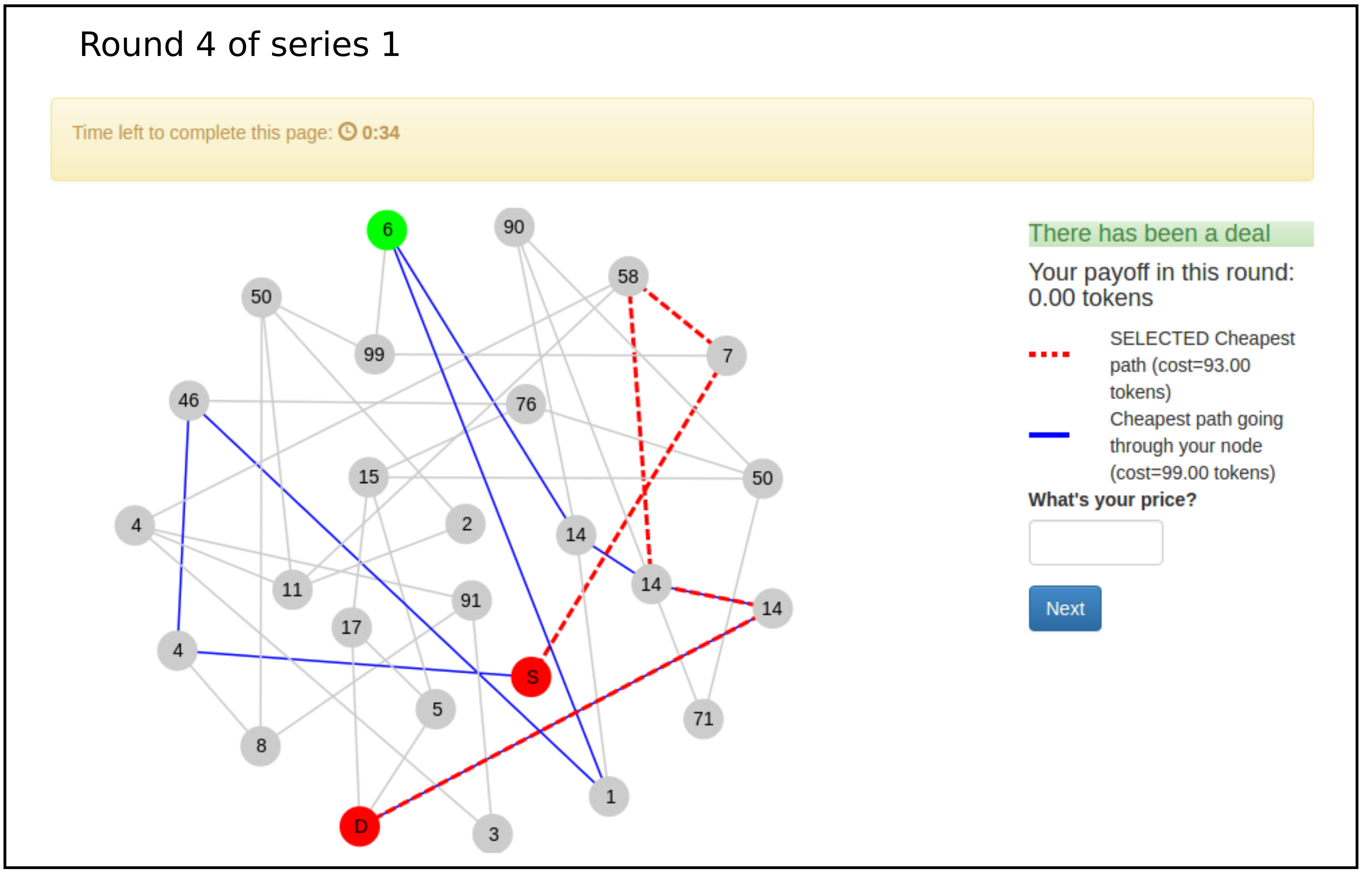

Please, click the below NEXT button to start:
[NEXT]

II. ADDITIONAL RESULTS

Here, we present additional results aimed to support the findings shown in the main text.

A. 50 nodes random network

The robustness of the findings shown in the main text against the size and connectivity of the network are explored through an additional experimental session in a random network of 50-nodes with $\langle k \rangle = 4$. Table S2 puts together the results corresponding to the random network with 50 nodes and those corresponding to 26-nodes networks. Although the larger network shows lower costs than the smaller ones (actually, for the 50-nodes network intermediation rents are close to zero), both the correlation between payoffs and SD-betweenness (Fig. S2) and the behavioral rule (Fig. S3) are verified in the network of 50 nodes, as will be discussed in the next paragraphs.

network	efficiency	price	price in CP	cost	profit	length
R 50	1	7.85	1.18	5.67	0.12	5.78
R 26	0.97	11.34	5.49	28.33	1.10	6.26
SW 26	0.68	18.10	13.16	76.52	2.38	7

TABLE S2. **Experimental results.** Efficiency (fraction of rounds in which the cheapest path cost was equal to or less than the threshold), and mean values of the price, price on the cheapest path, cost of the cheapest path, profit, and cheapest path length for each one of the three studied networks: random networks with 50 and 26 nodes (R 50, R 26) and small-world network with 26 nodes (SW 26).

Fig. 2 of the main text shows the prices, payoffs and frequencies on the cheapest path for all realizations. This result indicates a correlation between profits and the source-destination betweenness (sd_∞). To ensure that this result is not spurious, Fig. S2 shows how those variables correlate with the centrality in each network. It is clear that the same behavior is maintained when we look at the results for each network.

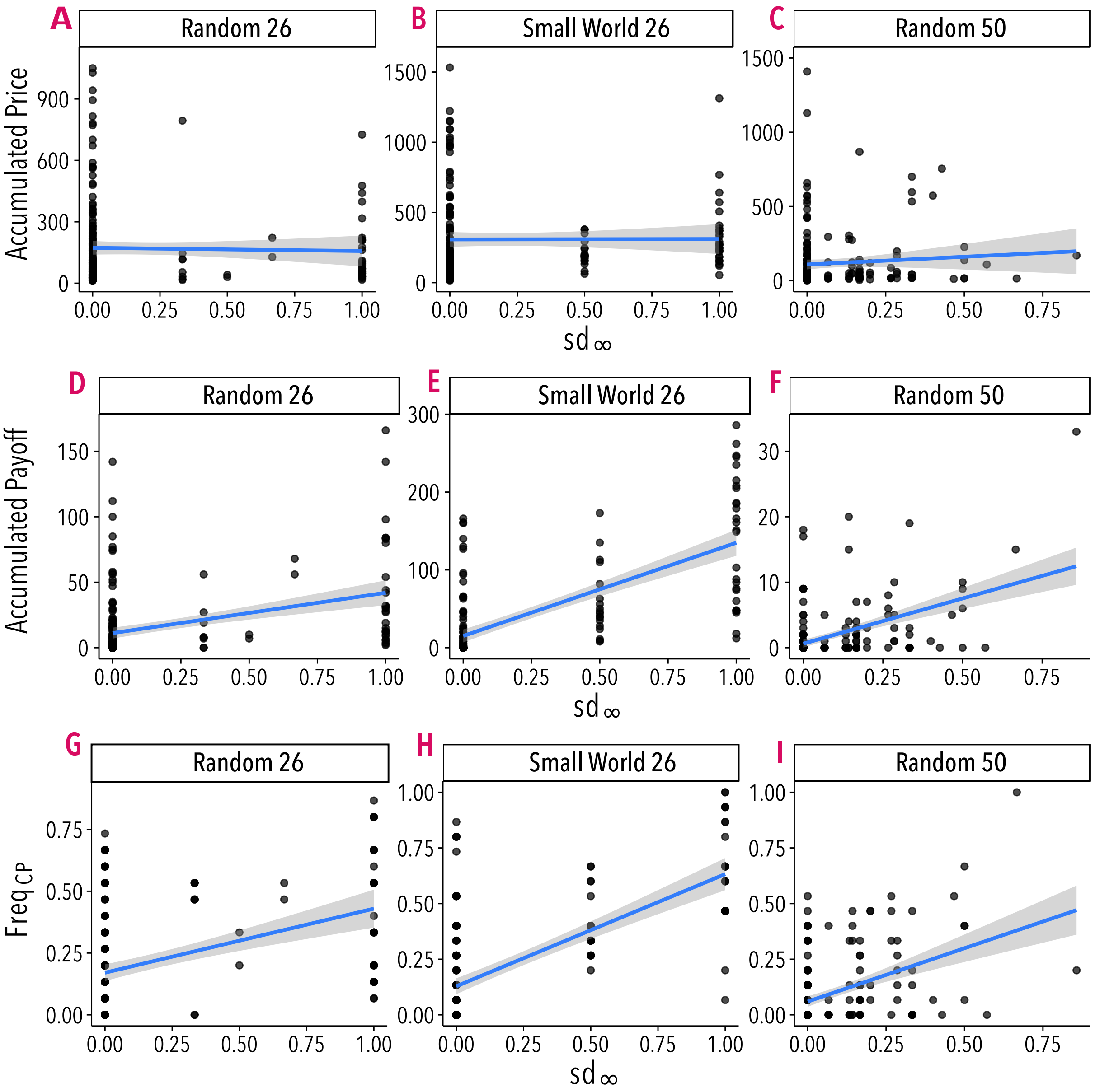

FIG. S2. **SD-betweenness determines payoffs but not posted prices. A-I**: Accumulated price (**A-C**), accumulated payoff (**D-F**) and frequency on the cheapest path (**G, I**) of participants during a series of 15 rounds as a function of the SD-betweenness sd_∞ for the Random 50 (**A,D,G**), Small World 26 (**B,E,H**), and Random 50 networks(**C,F,I**). Figure created using ggplot2 (v.3.2.1) [S3].

As an extension of Fig. 3 of main text, Fig. S3A shows the mean change in price for the cases where the participant was or was not on the cheapest path in the previous round, while Fig. S3B shows the probabilities to increase and to decrease the posted price conditioned to have been (Y) or not (N) on the cheapest path. In this figure, the results corresponding to the random network with 50 nodes have been added showing that, within the limitations of the current experiment, the behavioral rule according to which players increase their price if they were on the cheapest path in the previous round and decrease it otherwise is robust against the size and connectivity of the network.

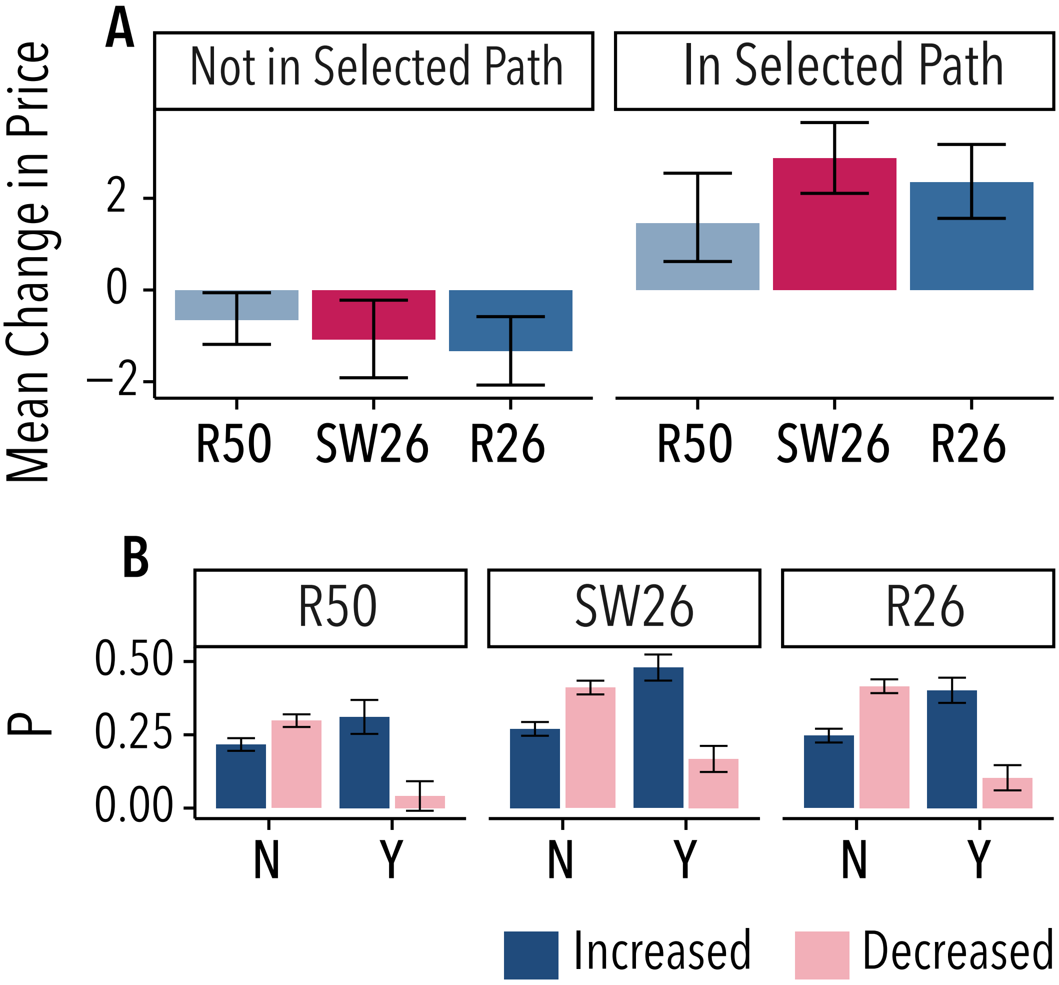

FIG. S3. **Extension of figure 3 of the main text with the 50-nodes random network. A:** Mean changes in the posted price for the studied networks: random network of 50 nodes (R50) and 26 nodes (R26), and small-world network of 26 nodes (SW26). The panel discriminates the cases in which the participant was (right) from those in which she was not (left) on the selected cheapest path in the previous round. **B:** Probability to increase (blue) and to decrease (pink) the posted price conditioned to have been (Y) or not (N) on the selected cheapest path, for each one of the studied networks. The error bars represent the 95% C.I. Figure created using ggplot2 (v.3.2.1) [S3].

B. Additional experimental results: Evolution of costs and prices

Fig. S4A shows the evolution of the cheapest path cost for each network. As described in the main text, the costs are significantly higher in the Small-World network than in the Random Network. Among the random networks considered, the costs are lower in the larger network (50 nodes) than in the smaller one (26 nodes). These higher costs in the SW network entail a lower efficiency, as can be seen in table S2. To deepen this issue, Fig. S4B represents the evolution of the mean price of participants on the cheapest path for each network. Note that the pattern is consistent with Fig. S4A, pointing out that the previous finding was not a consequence of different cheapest path lengths. To complement these results, Fig. S4C and S4D show, respectively, the evolution of the mean and median price posted by all the participants. As has been discussed in the main text, the effect of the network topology on prices (Fig. S4C, S4D) is not as clear as it is on costs and payoffs (Fig. S4A, S4B) since the topology mainly affects the probability for the nodes to be on the cheapest path more than the posted prices.

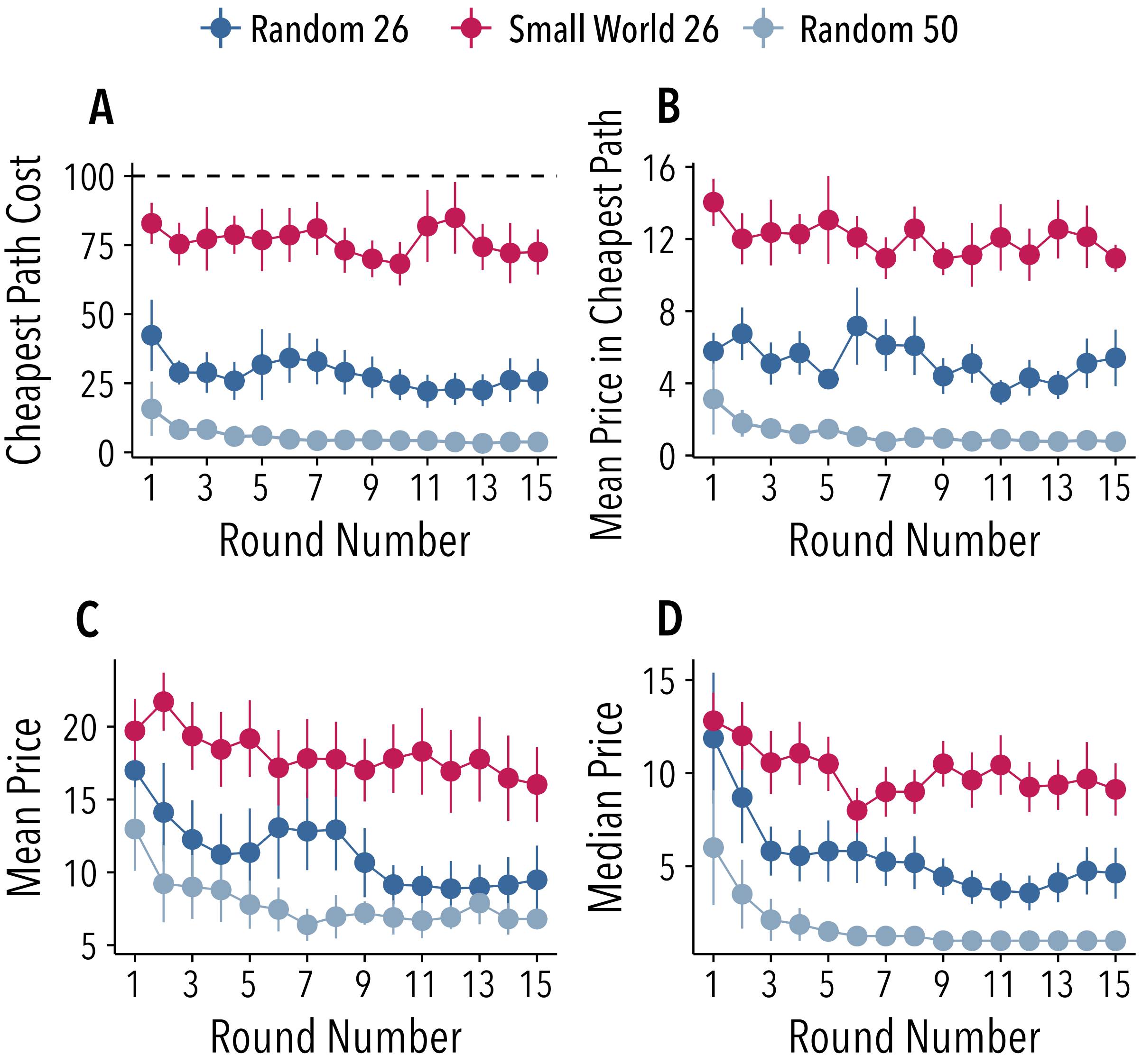

FIG. S4. **Evolution of costs and prices for each experimental network.** Cheapest path cost (**A**), mean price of nodes on the cheapest path (**B**), mean price (**C**), and median price (**D**) as a function of the round number. Each series of points corresponds to a given network: Random Network with 50 nodes (dark blue), Small-World with 26 nodes (magenta), and Random Network with 26 nodes (light blue). The error bars represent 1.96 × SEM. Figure created using ggplot2 (v.3.2.1) [S3].

C. Additional model results: Final costs and network metrics. Robustness.

1. Node-disjoint paths

In the game, nodes in a path between the source and the destination constitute a group, and all such groups can be seen as competing to represent the cheapest path. At the same time, individuals within those groups try to maximize their own profit, and as such are competing with other group members. Therefore, with a large number of paths there will be a large number of groups competing to be the cheapest path, with one caveat: paths do not constitute disjoint sets of nodes, which implies that nodes compete concurrently in a large number of groups. These conditions make the assessment of a good descriptor of the competition in a network a non-trivial task. Nonetheless, we can provide a lower bound estimator based on the minimum number of independent groups that can compete with each other using

the number of node-disjoint paths[1].

The results shown in Fig. 6 of the main text show that, when considering the behavioral rule found, the number M of node-disjoint paths is a particularly good indicator of the final trading costs. With more independent paths, more groups of different nodes can coordinate resulting in a cheaper cost. To illustrate the mechanism behind this relationship, we propose the following simple scenario wherein a formal relationship between the two variables can be demonstrated: i) all the node-disjoint paths are shortest paths and ii) all the nodes start with the same price. For this kind of scenario, we can see a clear relation between costs and M, as shown by Lemma 1. Indubitably, this lemma does not provide us a rule for how costs will change in all the possible networks. Nonetheless, it provides a useful insight into how the competition between paths for the cheapest path should be related to M.

Lemma 1. *Let us consider a graph G, a source S, a destination D, and identical initial prices across all nodes. Let us assume that nodes increase their posted prices by σ if they were located in the previous round in the cheapest path, otherwise decrease it by ρ. If there are M node-disjoint shortest paths of the same length between S and D, the cheapest path cost will increase indefinitely if only if*

$$\frac{\sigma}{\rho} > (M-1)$$

Proof. Let us consider an enumeration of the disjoint paths from S to D: $p_1, p_2, \ldots, p_M$. Let x be the initial posted price for all the nodes. Initially, a path (without loss of generality, p_1) will be selected, and nodes belonging to path p_1 will increase their price from x to $x+\sigma$, while nodes belonging to the rest of paths $(p_2, p_3, \ldots, p_M)$ will decrease their price to $x-\rho$. In the subsequent steps, the rest of the paths $(p_2, p_3, \ldots, p_M)$ will be selected until all the M paths will have been selected. At step M, the selected path will have a cost per node of $x-(M-1)\rho$, thus, its nodes will increase their price to $x+\sigma-(M-1)\rho$, which will be higher than x only if $\sigma > (M-1)\rho$. Note that, by the same reasoning, at step $M+1$ all the nodes located in $p_1, \ldots, p_M$ will have a cost of $x+\sigma-(M-1)\rho$. Therefore, the cost will increase indefinitely if and only if $\sigma > (M-1)\rho$. □

2. Average Path Length and Clustering Coefficient

Fig. 6 from the main text shows how trade costs scale with the average path length of the network. This result is not a consequence of cheapest paths length differences: the mean price of nodes on the cheapest path also correlates with the average path length, as shown in Fig. S5.

It is well known that small world networks differ from random networks with respect to clustering and average path length in different ways. Therefore, the clustering coefficient is also a natural candidate for capturing the differences in the cheapest path cost, as it is indeed the case as shown in Fig. S6. To check which of both observables is more connected with the network properties driving the differences in cost, we executed two linear regressions with final trade cost (i.e., on the cheapest path) as the dependent variable: one having the clustering coefficient as independent variables and one with the average path length as independent variables. The data considered are the results from simulations executed without the threshold, which are shown in Fig. 6 on the main text. As the slopes of both properties change with respect to M, we added an individual coefficient for each value of M obtained by multiplying the network property by a Kronecker delta (δ) dummy variable. The regression model with clustering coefficient (T) is shown in Eq. S1, and that with average path length (L) in Eq. S2. We restricted the data to $M \leq 3$, as for larger values final costs are mostly zero.

$$C_i = \beta_1 T_i \delta_{M_i 1} + \beta_2 T_i \delta_{M_i 2} + \beta_3 T_i \delta_{M_i 3} \tag{S1}$$

$$C_i = \beta_1 L_i \delta_{M_i 1} + \beta_2 L_i \delta_{M_i 2} + \beta_3 L_i \delta_{M_i 3} \tag{S2}$$

The results from the regression are displayed in Table S3 which shows that the model with average path length as regressors provides a better description of the final trade costs: the coefficient of determination when considering the average path length is $R^2(L) = 0.79$, while the model with the clustering coefficient achieves $R^2(T) = 0.57$.

---

[1] There is also an important practical fact to take into consideration: metrics based on counting all paths are unfeasible for relatively large and dense networks, as the number of paths grows exponentially. Fortunately, computing the maximum number of independent paths is reducible to the maximum flow problem, thus it can be computed in polynomial time.

	Model with Clustering Coefficient	Model with Average Path Length
Clustering Coefficient for $M = 1$	0.74^{***}	
	(0.00)	
Clustering Coefficient for $M = 2$	0.04^{***}	
	(0.00)	
Clustering Coefficient for $M = 3$	-0.09^{***}	
	(0.00)	
Average Path Length for $M = 1$		0.97^{***}
		(0.00)
Average Path Length for $M = 2$		0.20^{***}
		(0.00)
Average Path Length for $M = 3$		0.07^{***}
		(0.00)
R^2	0.57	0.79
Adj. R^2	0.57	0.79
Num. obs.	131148	131148
RMSE	0.66	0.46

$^{***}p < 0.001$, $^{**}p < 0.01$, $^{*}p < 0.05$

TABLE S3. **Coefficients of the statistical models.** Coefficients of the two statistical models, having the clustering coefficient as the independent variable (*left*), and having the average path length as the independent variable (*right*). The coefficients are standardized (centered and divided by their standard deviation). As the coefficients of clustering and average path length seem to vary with M, we consider it as a dummy variable. Slopes (clustering coefficient and average path length) are M-specific.

3. *Robustness of the model against the choice of initial conditions*

To validate the proposed model, we executed it by bootstrapping the initial prices from those obtained from the experiments. The results, shown in Fig. 5 of the main text, indicate that costs and mean prices in the cheapest path are higher in small-world networks than in random networks, in agreement with the experimental results.

Furthermore, to study the effect of initial prices on the model, we have executed the algorithm with random initial prices, disregarding the data from experiments and then making the model self-consistent. To this end, we have taken the initial prices according to a Poissonian distribution ($\lambda = 10$). The results for the Poissonian distribution for the initial prices are displayed in Fig. S7, being compatible with those corresponding to bootstrapping displayed in Fig. 5 of the main text, therefore highlighting the robustness of the model against the choice of initial conditions and validating the effects discussed in the main text about the network structure influence on prices and costs.

[S1] Daniel L Chen, Martin Schonger, and Chris Wickens, "oTree—An open-source platform for laboratory, online, and field experiments," Journal of Behavioral and Experimental Finance **9**, 88–97 (2016).

[S2] Duncan J Watts and Steven H Strogatz, "Collective dynamics of small-world networks," Nature **393**, 440 (1998).

[S3] Hadley Wickham, *ggplot2: Elegant Graphics for Data Analysis* (Springer-Verlag New York, 2016).

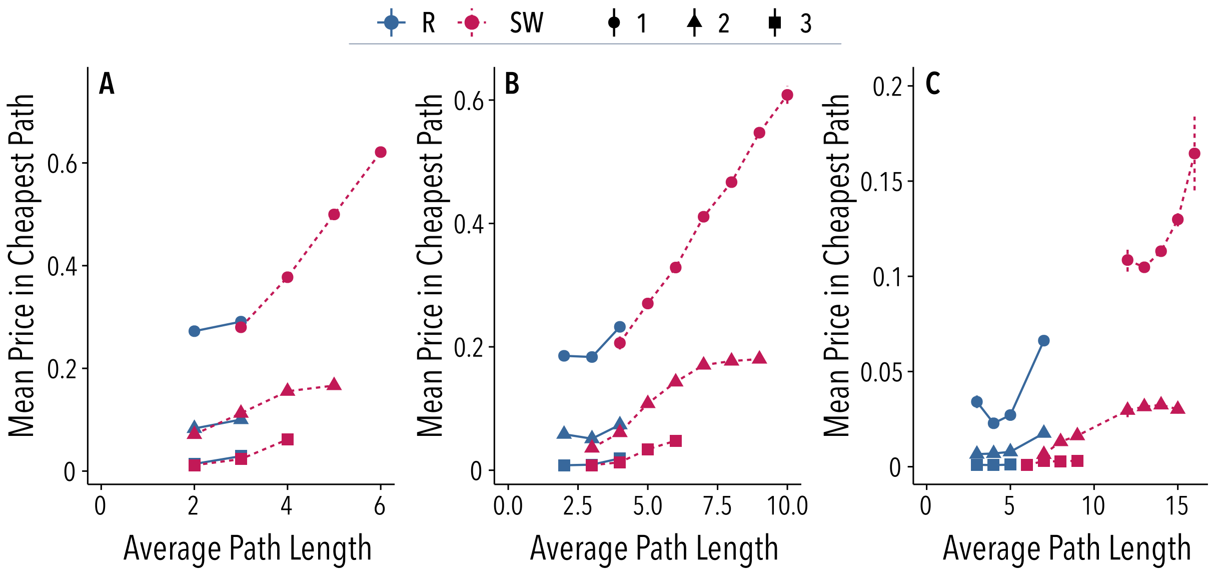

FIG. S5. **Numerical results of the model.** Average price of nodes on the cheapest path after a period of 10^4 rounds as a function of the mean path length of the network. Each panel corresponds to a different network size: 26 (**A**), 50 (**B**), and 1000 (**C**) nodes. Different colors correspond to different network models: random (blue) and small-world (magenta). Different simbols correspond to different values of the number M of disjoint paths: $M = 1$ (circles), 2 (triangles), and 3 (squares). For each configuration, there were generated 10000 networks of each size according to the Watts-Strogatz algorithm [S2] with $p = 0.1$ (SW) and $p = 1$ (R), and average degree from 2 to 10. The increment/decrement ratio was fixed to the experimental value ($\sigma/\rho = 2.4$). Figure created using ggplot2 (v.3.2.1) [S3].

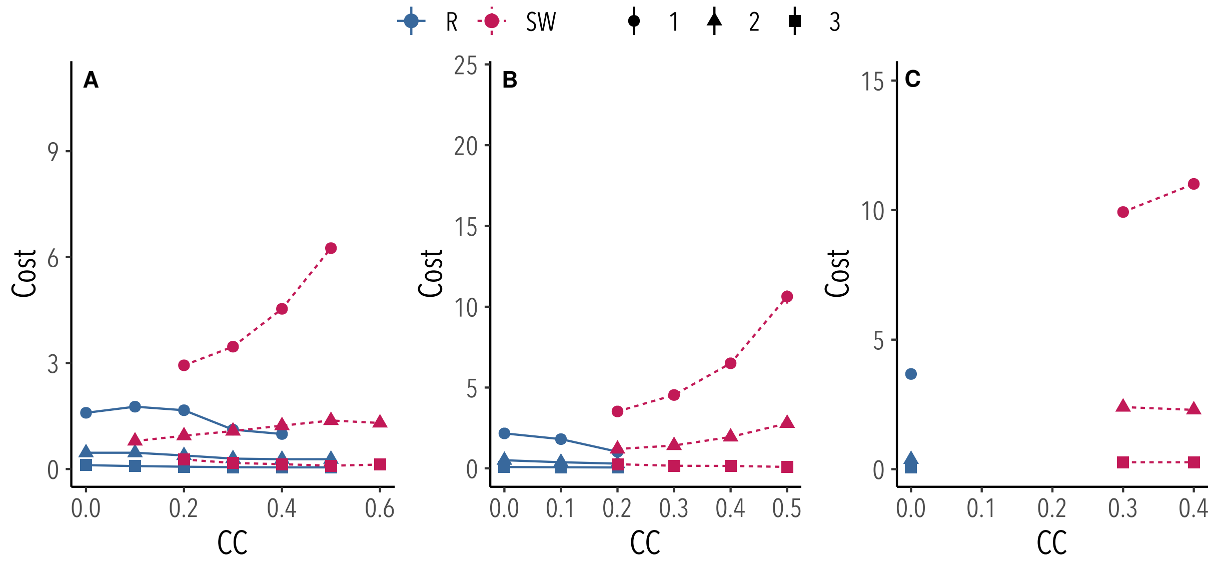

FIG. S6. **Numerical results of the model.** Average cost of the cheapest path after a period of 10^4 rounds as a function of the network clustering coefficient. Each panel corresponds to a different network size: 26 (**A**), 50 (**B**), and 1000 (**C**) nodes. Different colors correspond to different network models: random (blue) and small-world (magenta); while different symbols correspond to different values of the number M of disjoint paths: $M = 1$ (circles), 2 (triangles), and 3 (squares). For each configuration, there were generated 10000 networks of each size, according to the Watts-Strogatz algorithm [S2] with $p = 0.1$ (SW), $p = 1$ (R), and average degree from 2 to 10. The increment/decrement ratio was fixed to the experimental value ($\sigma/\rho = 2.4$). Figure created using ggplot2 (v.3.2.1) [S3].

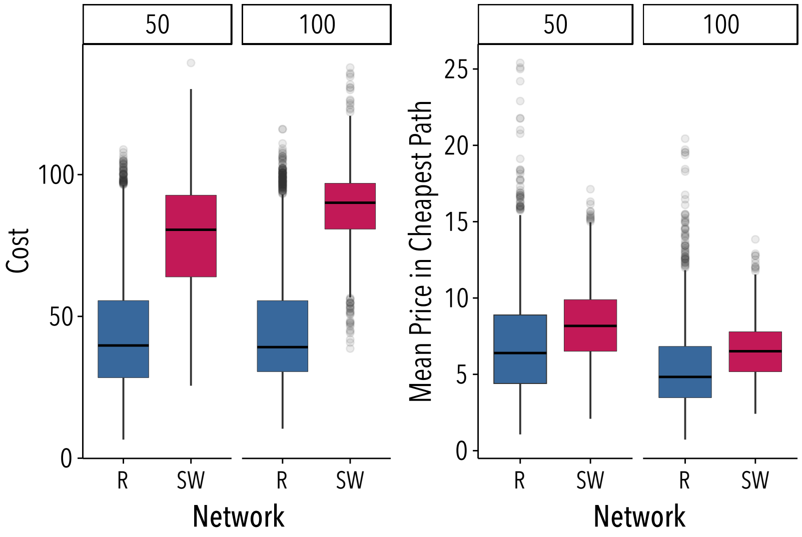

FIG. S7. Numerical results of the model for networks with 50 and 100 nodes. Results shown are for 100 executions with 15 rounds for each network and source-destination pair, excluding the first round. Initial prices are taken from a Poissonian distribution ($\lambda = 10$). Other values are $\sigma = 2.60$ and $\rho = 1.2$. Figure created using ggplot2 (v.3.2.1) [S3].
